# A new BWO-based RGB vegetation index and ensemble learning strategy for the pests and diseases monitoring of CCB trees using unmanned aerial vehicle

**DOI:** 10.3389/fpls.2024.1464723

**Published:** 2024-12-11

**Authors:** Keliang Hu, Junchen Liu, Hai Xiao, Qiangguo Zeng, Jun Liu, Lei Zhang, Man Li, Zhihui Wang

**Affiliations:** ^1^ School of Informatics, Hunan University of Chinese Medicine, Changsha, China; ^2^ AI TCM Lab Hunan, Changsha, China; ^3^ Tianjin Institute of Surveying and Mapping Co., Ltd., Tianjin, China; ^4^ The Second Surveying and Mapping Institute of Hunan Province, Changsha, China

**Keywords:** pests and diseases monitoring, Beluga Whale Optimization algorithm, BWO-based vegetation index, BWO-based ensemble strategy, unmanned aerial vehicle

## Abstract

**Introduction:**

The Cinnamomum Camphora var. Borneol (CCB) tree is a valuable timber species with significant medicinal importance, widely cultivated in mountainous areas but susceptible to pests and diseases, making manual surveillance costly.

**Methods:**

This paper proposes a method for detecting CCB pests and diseases using Unmanned aerial vehicle (UAV) as an advanced data collection carrier, capable of gathering large-scale data. To tackle the high cost and challenging data processing issues associated with traditional hyper-spectral/multi-spectral sensors, this method only relies on UAV visible light RGB bands. The process first involves calculating and normalizing 24 visible light vegetation indices from the UAV RGB images of the monitoring area, along with the original RGB bands. To account for the collinearity relationship between indices, the random forest variable importance and correlation coefficient iterative analysis algorithm are employed to select indices, retaining the most important or lowest collinearity multiple vegetation indices. Subsequently, the Beluga Whale Optimization (BWO) algorithm is utilized to generate a new vegetation index, which is then combined with the multi-threshold segmentation method to propose a BWO-weighted ensemble strategy for obtaining the final pests and diseases detection results.

**Results and discussion:**

The experimental results suggest that the new BWO-based vegetation index has a higher feature expression ability than single indices, and the new BWO-based ensemble strategy can yield more accurate detection results. This approach provides an effective means for low-cost pests and diseases detection of CCB trees.

## 1 Introduction

The Cinnamomum Camphora var. Borneol (CCB) is an esteemed medicinal timber species gaining popularity, which stands out in the botanical realm due to its profuse presence of borneol, a naturally occurring camphor with a distinct fragrance and therapeutic properties. It is extensively utilized in Traditional Chinese Medicine and is hailed as a quintessential example of aromatic medicinal herbs. The CCB tree demands stringent growth conditions and is very particular, thriving only under specific climatic and soil circumstances. It frequently faces threats from a variety of pests and diseases, which significantly impact its growth and the efficiency of its resource utilization. Consequently, the rapid and precise monitoring of pests and diseases affecting the CCB tree is essential for guaranteeing its yield and maintaining its medicinal efficacy.

Advancements in remote sensing technology have made it possible to utilize various aerial and space-based remote sensing technologies for pests and diseases monitoring, which is now a crucial method (Li et al., 2010). In the field of pests and diseases monitoring, satellite remote sensing technology has been applied for a long time and has achieved impressive results. By analyzing spectral information in multi-spectral images, a wide range of forestry pests and diseases can be effectively identified and monitored. However, satellite remote sensing technology has limitations, such as temporal delays in monitoring results due to long revisit cycles, restrictions in imaging conditions, atmospheric interference, and topographical undulations. Although high-resolution satellite data, such as IKONOS, QuickBird, and WorldView, can provide precise remote sensing data at the meter or even sub-meter level, they are expensive to acquire, have slow update speeds, and may not have sufficient spatial resolution for precise identification at the tree species level.

In recent years, the rapid evolution of unmanned aerial vehicle (UAV) has elevated it to a crucial platform for terrestrial monitoring with inherent speed, convenience, and effectiveness (Wu et al., 2024). UAV remote sensing swiftly captures a diverse array of images, including high-definition, multi-temporal, multi-angular, multi-spectral, and hyper-spectral images, even in intricate terrain. This technology significantly supports forest pests and diseases surveillance efforts in specific regions. However, as image resolution improves, it simultaneously presents a richer source of information, which necessitates the development of algorithms that can maintain precision and processing speed despite these increased data complexities.

The fundamental principle of using UAV technology for monitoring forest pests and diseases lies in the fact that plants, when exposed to pest or disease stress, typically display distinct stress symptoms or damage, resulting in alterations to their spectral reflectance that can be captured by UAV sensors. By utilizing multi-spectral or hyper-spectral UAV sensors, researchers have acquired high-resolution UAV data, capitalizing on plant spectral characteristics to create artificial intelligence-related algorithms for pest and disease monitoring, with satisfactory detection accuracy. For example, Park et al. (2021) utilized a multi-spectral camera-equipped UAV to capture high-resolution multi-spectral aerial images, and applied a multi-channel CNN-based object detection technique to detect pine wilt disease-infected trees, achieving impressive detection results. Run Yu et al. (2021) integrated UAV multi-spectral images with target detection algorithms to monitor pine wilt disease incidence, providing technical support for its control. Kim et al. (2015) extracted pine wilt disease-affected trees using time-series hyper-spectral aerial images and analyzed their distribution characteristics. Zeng et al. (2023) achieved a maximum recognition rate of 93.2% for early detection of rubber tree white powder disease using UAV multi-spectral remote sensing. Sahin et al. (2023) utilized a multi-spectral camera and CRF-enhanced U-Net for weed identification, achieving an average recognition rate of 88.3% for soil, crops, and weeds. Luis Pádua et al. (2020) utilized UAV multi-spectral images and a random forest algorithm to select crown height model (CHM) features for cork oak tree pest and disease monitoring, with the highest overall accuracy of 91% in September and the lowest of 76% in May. Sivakumar et al. (2020) trained and evaluated convolutional neural network models based on target detection for pest detection in low-altitude UAV images, concluding that the Faster RCNN model performed best in detecting late-stage weeds in soybean fields.

Multispectral/hyperspectral sensors have more spectral channels, which can capture finer spectral bands, thus obtaining more detailed material spectral information. This is helpful for analyzing land features, studying various spectral matching models, and is widely used in military reconnaissance, mineral exploration, environmental monitoring, precision agriculture, medical diagnosis, and other fields. However, their cost is high, the system is more complex, and they require a higher level of professional operation. Specialized data processing techniques are needed to analyze the large amount of spectral data. Moreover, while providing high spectral resolution, they may sacrifice some spatial resolution. Therefore, the cost of obtaining high-resolution multispectral/hyperspectral UAV image data is high and suitable for fields that require higher precision and more challenging identification tasks. Ordinary visible light sensors typically have only three bands (red, green, blue). Due to the limited number of bands, the amount of information provided is limited and mainly used for basic image capture and some simple environmental monitoring. However, they usually have a higher spatial resolution, suitable for capturing clear images. Because of their lower cost, simpler system, easy mass production and application, and relatively easier data processing, many scholars use lower-cost high-resolution RGB image data instead of high-cost multispectral/hyperspectral images. These RGB data are also converted into various vegetation indices, such as VEG, CIVE, and others, to facilitate the detection of crop pests and diseases. In contrast to satellite remote sensing, the spatial resolution of UAV remote sensing can achieve levels of decimeters or even centimeters, substantially diminishing the influence of mixed pixels on the accuracy of estimations. However, the payload capacity of small, low-altitude UAV remote sensing platforms is restricted, often precluding the use of high-precision professional instruments, and typically relies on visible light cameras, which results in a deficiency of near-infrared information. To mitigate this issue, researchers have exploited the trough-peak-trough features of vegetation reflectance within the visible light spectrum to create diverse visible light vegetation indices. These indices have been applied in studies related to vegetation information extraction, leaf greenness content estimation, and more. For instance, Wang Xiaojun et al. (2015) successfully distinguished between vegetation and non-vegetation in UAV images using visible light vegetation indices; Hunt Jr. et al. (2005) utilized UAV visible light vegetation indices to forecast the yield of corn and soybeans; Schmidt et al. (2017) employed a UAV-based species identification system for rangeland plants (with a spatial resolution of 3 m) based on RGB images (with a resolution of 30 cm) to conduct distribution monitoring of needlegrass species through a supervised classification support vector machine approach, using RGB images as ground reference data. Bryson Mitch et al (Bryson et al., 2010; Garcia-Ruiz et al., 2013) have applied visible light low-altitude UAV images to monitor yellow dragon disease in crops and have widely implemented this in precision agriculture practices.

Preliminary research has revealed that the collective performance of various vegetation indices offers enhanced stability and dependability over individual indices when monitoring crop pests and stresses. The simplest approach to integrating multiple vegetation indices is by using a weighted summation, yet the determination of appropriate weights presents a significant challenge in this field. To address the constraints of conventional methods, this paper incorporates the BWO (Beluga Whale Optimization) algorithm into the weighted segmentation process of vegetation indices, with the goal of achieving accurate monitoring of plant pests and diseases. The BWO algorithm, an optimization method inspired by biological behavior, boasts global search capabilities and rapid convergence, showcasing its effectiveness in tackling optimization challenges. The CCB trees typically grow on mountains, and the conventional pest and disease monitoring methods that rely on manual inspection are time-consuming, labor-intensive, and inefficient, posing safety risks to personnel. Drones offer an aerial perspective for inspecting large areas of CCB trees, effectively addressing many of the limitations of conventional manual inspections. The aim of this study is to apply the BWO algorithm for the weighted combination of vegetation indices, integrating diverse threshold segmentation methods to detect pest and disease-infested areas in CCB Trees. The red (R), green (G), and blue (B) color bands along with 24 additional vegetation indices are chosen for BWO-based weighting to create a novel vegetation index. These indices provide essential information on the physiological state and growth conditions of the plants. A rational weighting of the CCB Tree’s vegetation index can boost the precision and efficiency of pest and disease monitoring, thus providing a scientific basis and technical support for the prompt identification and prevention of pests and diseases in CCB Trees.

The contribution of this paper can be summarized as follows:

1. The paper presents a novel method for generating a new vegetation index utilizing the BWO algorithm. This method innovatively amalgamates data from several vegetation indices across different aspects, solely with the use of RGB images, to develop a novel vegetation index that surpasses individual indices in detecting pests and diseases with higher accuracy.
2. The paper presents a method for identifying pests and diseases in plants by combining multiple threshold segmentation methods with a newly developed vegetation index. By employing a simple ensemble strategy based on majority voting, this technique shows improved detection performance over individual threshold segmentation techniques and standalone vegetation indices.
3. The paper proposes an ensemble strategy optimized by the BWO algorithm, which leverages a variety of threshold segmentation methods along with a novel vegetation index. This approach delivers a more reliable enhancement in the detection accuracy of pests and diseases by dynamically adjusting the weights of each threshold segmentation technique.
4. Considering the multicollinearity among various vegetation indices derived from RGB data, this study applies Random Forest Variable Importance and Correlation Coefficient Iterative Analysis to evaluate the collinearity among multiple vegetation indices. The results indicate that using a selected subset of these indices can significantly reduce computational complexity without compromising detection accuracy, by accounting for the multicollinearity among them.

The organization of this paper is as follows: In section 2, the study area and the data used are introduced. Section 3 outlines the methodologies employed, which include 24 different vegetation indices, Random Forest Variable Importance, Correlation Coefficients Iterative Analysis using, Voting Ensemble Approach, and the BWO Ensemble Strategy. Section 4 provides the experimental outcomes, confirming the efficacy of the BWO method. The paper concludes with final remarks in Section.

## 2 Materials and methods

### 2.1 Study area overview

The study area is located in the CCB Plantation Base in Xinhuang County, Huaihua City, Hunan Province. Xinhuang Dong Autonomous County (hereinafter referred to as Xinhuang County) belongs to the mid-subtropical monsoon humid climate, with distinct four seasons, warm and humid, short cold period, long frost-free period, abundant rainfall, with an average annual temperature of 16.6°C, an average annual precipitation of 1160.7 mm, and a total sunshine between 1014.5 and 1590.2 hours. The annual frost-free period is 297.4 days. The geographical location is between 108°47’13” - 109°26’45” east longitude and 27°4’16” - 27°29’58” north latitude. It is located in the western part of southwestern Hunan, under the jurisdiction of Huaihua City, Hunan Province, at the end of the extension of the Miaoling Mountains in the Yunnan-Guizhou Plateau, mainly with mountainous topography, with a forest coverage rate of 67.5%, of which the planting area of CCB trees reaches 18 km, which is the leading variety of traditional Chinese medicine in the county. The origin is in the gentle slope land below 800 meters above sea level and with a slope of less than 25°; the soil layer is loose, and the soil type is yellow soil or red soil. The pH value is 5.5 to 7.0, the soil depth is more than 50 cm, the drainage and air permeability are good, and the organic matter content is ≥1.0%. The superior geographical and climatic conditions, suitable temperature and precipitation, and vast mountain resources provide an ideal growth environment for the growth of CCB trees, and provide sufficient raw materials for the production and development of Xinhuang CCB.

### 2.2 UAV data collection

**Figure 1:**
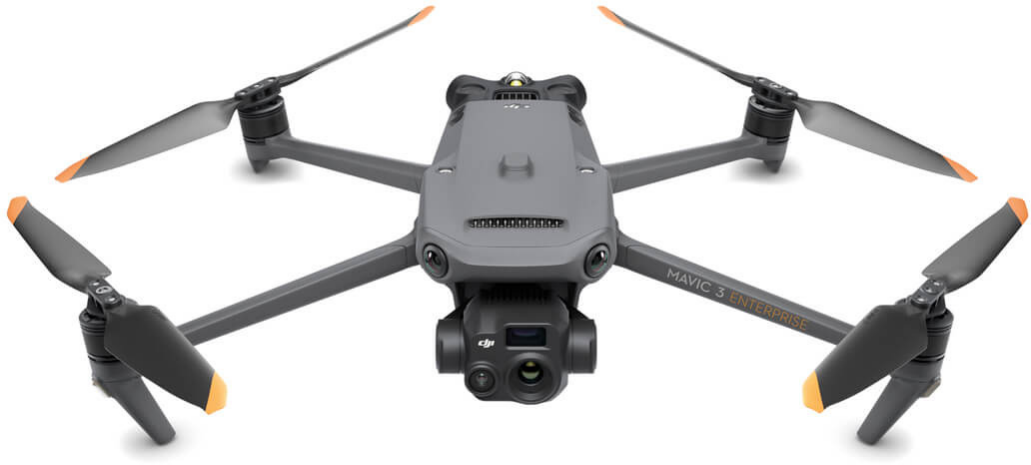
DJI Mavic 3M UAV.

The data collection in this study was carried out using a DJI Mavic 3M UAV, as depicted in **Figure 1**.

This professional UAV is equipped with a 20-megapixel visible light camera and four 5-megapixel multispectral cameras, facilitating high-precision aerial surveys, crop growth monitoring, and natural resource investigations. The UAV also utilizes multispectral sensors to capture solar radiation data, which is stored in image files, improving the accuracy and consistency of NDVI (Normalized Difference Vegetation Index) results. Equipped with RTK (Real-Time Kinematic) technology, it provides centimeter-level precision positioning. The UAV’s specifications include a bare weight of 951 grams (including propellers and RTK module), a maximum takeoff weight of 1050 grams, dimensions of 347.5mm * 283mm * 139.6mm. Its flight time is up to 43 minutes, and the longest hovering time is 37 minutes, allowing a single flight to cover up to 3000 acres for mapping tasks. The UAV offers adjustable flight speed and stable flight, with the capability to hover precisely, making it suitable for repeated, multi-scale, and high-resolution data acquisition of crop pests and diseases stress at specific points.

**Figure 2:**
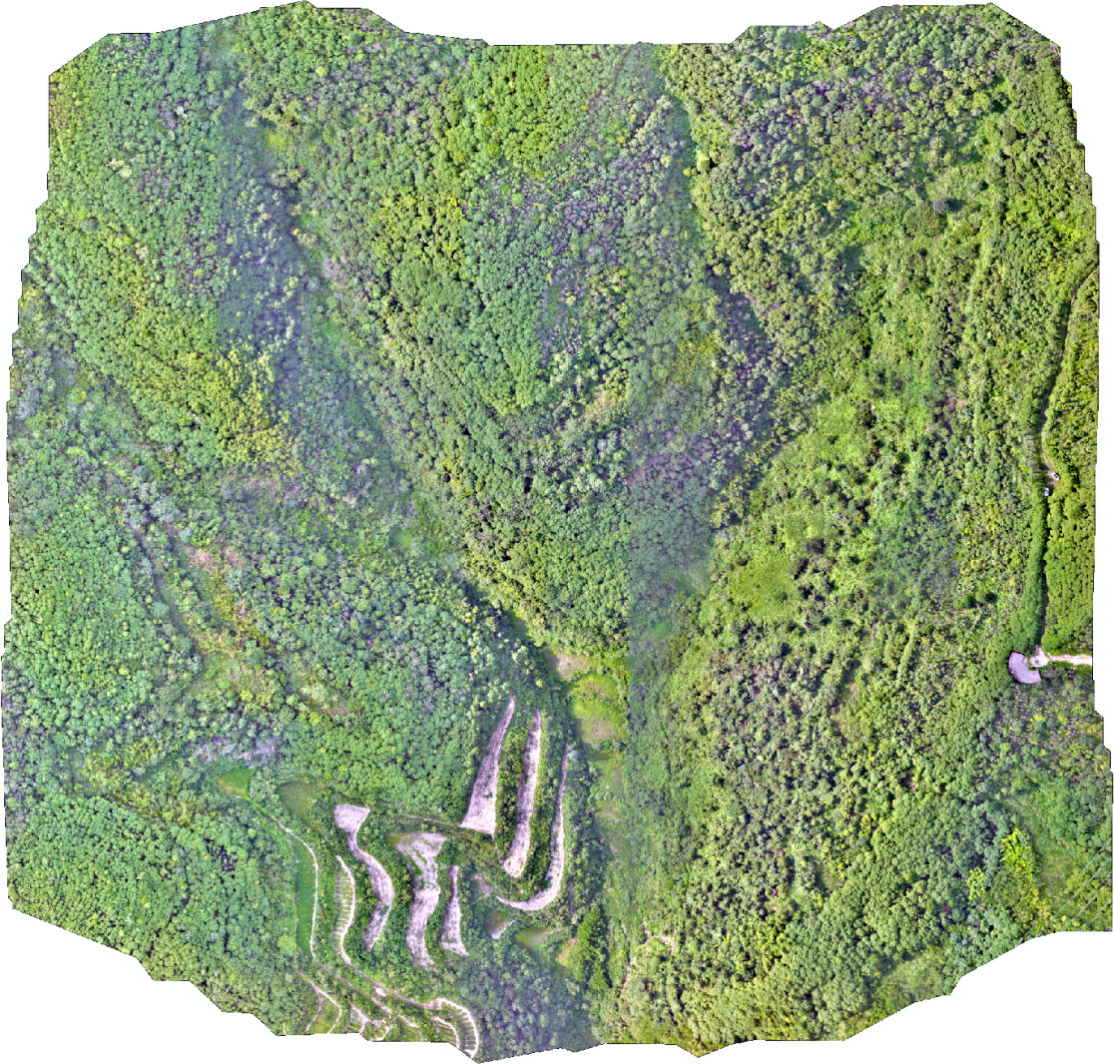
Mosaic RGB image of the study area by UAV.

In Xinhuang County, a severely pests and diseases affected area within the CCB plantation base was chosen for UAV multispectral and RGB visible light image acquisition. The aerial imaging mission took place on July 28, 2023, at 3:00 PM local time, with ideal weather and a soft breeze. The UAV operated in a constant-altitude patrol mode, maintaining a downward-facing camera lens. During the flight, the conditions were favorable, with an altitude of approximately 80 meters and intermittent hovering for image acquisition. Due to relaxed specifications for spectral range and pixel center alignment, no radiometric calibration was applied to the images. The captured visible light images consisted of three fundamental bands: red, blue, and green. Following preprocessing with DJI-Terra software, the RGB data generated a seamless mosaic of the entire region, as depicted in **Figure 2**, where darker shadow areas denote the mountain’s rear slopes.

**Figure 3:**
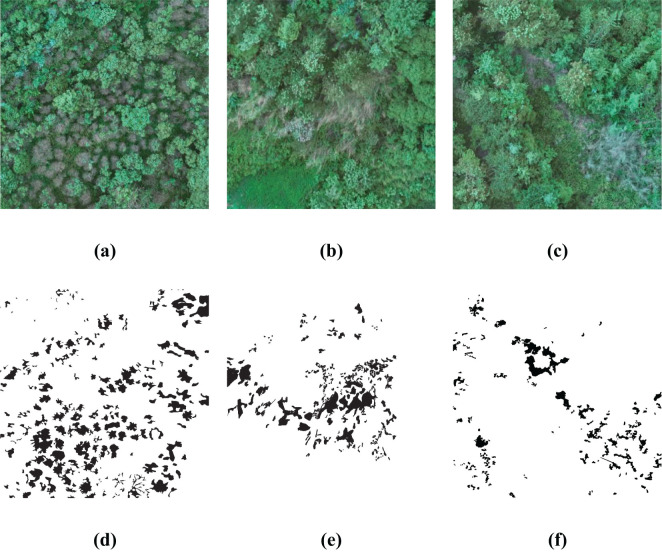
Sub-images of the study area and the corresponding ground truth. **(A–C)** are the original RGB images; **(D–F)** are the corresponding ground truth.

As described in following **Figure 3**, three sub-regions within the image are chosen for algorithmic study, which clearly show plants damaged by pests and diseases. By manually delineating the exact locations of the affected plants, the gray scale values for these regions are set to 0, whereas the gray scale values for the healthy plant areas are set to 255. As a result, a binary ground truth image is created, which can be used for both training the algorithm and assessing accuracy metrics.

## 3 Methods

### 3.1 General methodology

**Figure 4:**
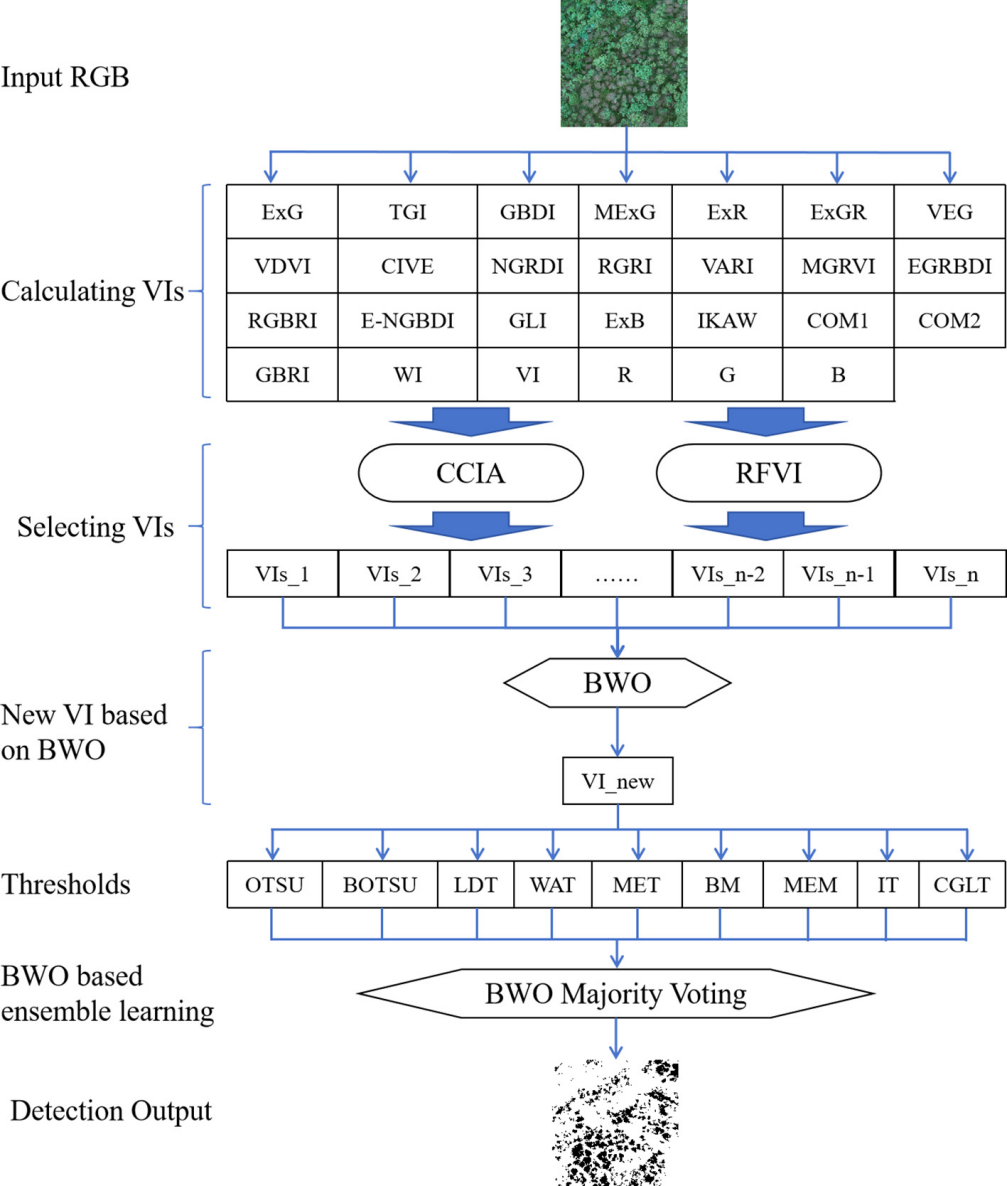
Overall technical flowchart.

Based on a variety of RGB-derived vegetation indices, this paper proposes a new vegetation index based on BWO and a new ensemble learning strategy to achieve precise detection of trees with pests and diseases. The specific technical route is shown in **Figure 4** below.

To begin with, high-resolution RGB image data was collected using an UAV and underwent essential preprocessing for quality assurance, including image matching, image mosaic, image dodging and so on. Following this, various vegetation indices were extracted from the images, including Normalized Green-Red Difference Index (NGRDI), Green Leaf Index (GLI), Red-Green Ratio Index (RGRI), and Excess Green Index (ExG), along with others, to describe vegetation coverage and health status. These computed indices were subsequently optimized using random forest variable importance and correlation coefficients iterative analysis. The BWO algorithm was employed in a small region of the image, with the Critical Success Index (CSI)s index after single-threshold segmentation set as the optimization objective, to construct a new vegetation index based on the optimization algorithm. The optimized weights were subsequently applied to the entire image. Ultimately, based on the ensemble threshold approach, a new ensemble strategy based on BWO was proposed, generating the final pests and diseases detection results.

### 3.2 An overview of 24 vegetation indices

Currently, vegetation indices are mainly based on visible and near-infrared wavelengths, including the NDVI and the Ratio Vegetation Index (RVI). The remote sensing imagery necessary for these indices is usually characterized by high procurement expenses, limited real-time capabilities, and a coarse spatial resolution. In this study, 24 different vegetation indices were computed from visible light imagery obtained via UAV. By combining these indices with the original red, green, and blue (R, G, B) images, their potential application was investigated in the surveillance of pests and diseases affecting CCB trees.

The mathematical formulas for calculating indices within the visible light bands are detailed in **Table 1**.

**Table 1:** Visible vegetation index.

VIs	formula	Reference
Eexess green index (ExG)	2∗green−red−blue	(Woebbecke et al., 1995)
Triangular greenness index (TGI)	green−0.39∗red−0.61∗blue	(Marques et al., 2021)
Green-blue difference index (GBDI)	green−blue	(Dai et al., 2020)
Modified excess green index (MExG)	1.26∗green−0.884∗red−0.311∗blue	(Hamuda et al., 2016)
Excess green index (ExR)	1.4∗red−green	(Meyer and Neto, 2008)
Excess Green minus Excess Red (ExGR)	ExG−ExR	(Meyer et al., 2004)
Vegetativen (VEG)	$green/(red^{a}-blue^{1-a}),a=0.667$	(Hague et al., 2006)
Visible-band difference vegetation index (VDVI)	(2∗green−red−blue)/(2∗green+red+blue)	(Liu. et al., 2020)
Color Index of Vegetation Extraction (CIVE)	0.441∗r−0.811∗g+0.385∗b+18.78745	(Kataoka et al., 2003)
Normalized Green-red Difference Index (NGRDI)	(green−red)/(green+red)	(Hunt et al., 2005)
Red Green Ratio Index (RGRI)	red/green	(Verrelst et al., 2008)
Visible Atmospherically Resistant Index (VARI)	(green−red)/(green+red−blue)	(Gitelson et al., 2003)
Modify Green-Red Vegetation Index (MGRVI)	$\left(green^{2}-red^{2})/(green^{2}+red^{2}\right)$	(Bendig et al., 2015)
Excess green-red-blue difference index (EGRBDI)	$\left({\left(2*green\right)}^{2}-red*blue)/({\left(2*green\right)}^{2}+red*blue\right)$	(Gao et al., 2020)
Red-Green-Blue Ratio Index (RGBRI)	$\left(green^{2}-red*blue)/(green^{2}+red*blue\right)$	(Zhao et al., 2019)
Enhance Normalized Red-Blue Difference Index (E-NGBDI)	$\left(green^{2}-blue^{2})/(green^{2}+blue^{2}\right)$	(Zhou and Zhu, 2016)
Green leaf index (GLI)	(2∗g−r−b)/(2∗g+r+b)	(Louhaichi et al., 2001)
Excess blue index (EXB)	(1.4*blue−green)/(green+red+blue)	(Mao et al., 2003)
kawashima index (IKAW)	(red−blue)/(red+blue)	(Kawashima and Nakatani, 1998)
1 (COM1)	ExG+CIVE+ExGR+VEG	(Guijarro et al., 2011)
2 (COM2)	0.36∗ExG+0.47∗CIVE+0.17∗VEG	(Guerrero et al., 2012)
GBRI	blue/red	(Sellaro et al., 2010)
Woebbecke index (WI)	(green−blue)/(red−green)	(Woebbecke et al., 1995)
VI	(2∗g−r−b)−(1.4∗r−g)	(Meyer and Neto, 2008)

where the red, green, and blue bands (R, G, B) correspond to pixel values of their respective wavebands, while r, g, and b signify normalized values for each.

**Figure 5:**
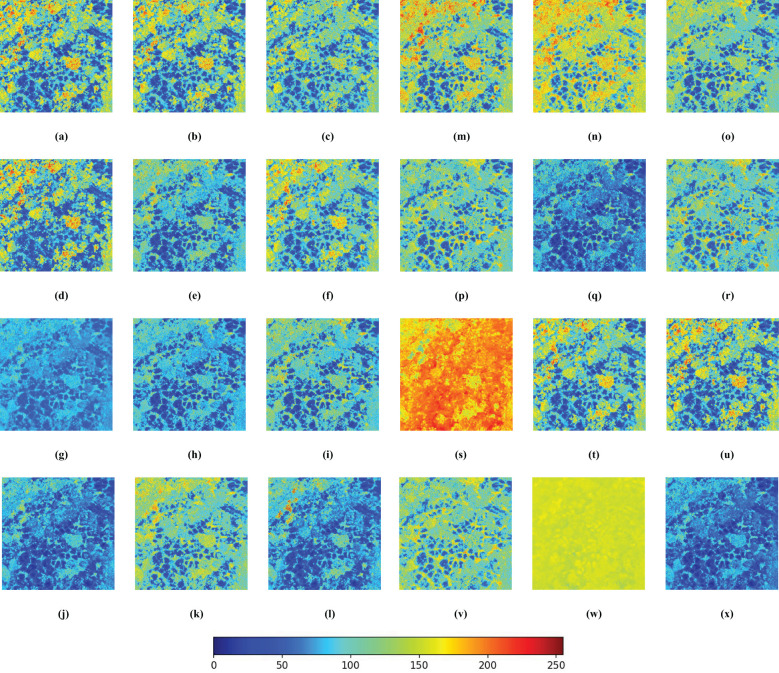
Pseudo-color images of 24 RGB-derived vegetation indices. The more blue a color is, the higher the probability that the tree is affected by pests and diseases; conversely, the more red it is, the healthier the tree is. **(A)** ExG; **(B)** TGI; **(C)** GBDI; **(D)** MExG; **(E)** ExR; **(F)** ExGR; **(G)** VEG; **(H)** VDVI; **(I)** CIVE; **(J)** NGRDI; **(K)** RGRI; **(L)** VARI; **(M)** MGRVI; **(N)** EGRBDI; **(O)** RGBRI; **(P)** E-NGBDI; **(Q)** GLI; **(R)** ExB; **(S)** IKAW; **(T)** COM1; **(U)** COM2; **(V)** GBRI; **(W)** WI; **(X)** VI.

Each vegetation index represents a distinct plant response to specific environmental conditions. Due to the use of normalized RGB bands in certain indices, leading to inconsistent units, this paper normalized the 24 vegetation indices derived from RGB images and created grayscale images for uniformity. All subsequent analyses were carried out on these normalized images, as depicted in **Figure 5**. The pseudo-color image results reveal that indices such as the GLI, RGRI, and EGRBDI tend to be more sensitive to plants suffering from pests and diseases. The ExGR stands out in identifying healthy vegetation. On the other hand, indices like the IKAW and WI may not be as efficient in reflecting plant health. By integrating the responses of these indices to the healthy and unhealthy plants, the accuracy of pests and diseases monitoring can be significantly improved. This holistic approach leverages the complementary nature of diverse vegetation indices, facilitating more precise detection and evaluation of pests and diseases impacts in remote sensing surveillance.

### 3.3 Nine threshold segmentation methods

Threshold segmentation is a region-based image segmentation technique that categorizes image pixels into various groups. This technique, known as image threshold segmentation, is a traditional and frequently used method in image segmentation, and it has become the most basic and widely applied segmentation technique due to its simplicity, low computational cost, and stable performance. The objective of image threshold is to partition the set of pixels according to grayscale levels, resulting in subsets that form regions corresponding to real-world objects. These regions have consistent properties within them, while adjacent regions do not share such consistency. This partitioning is achieved by selecting one or more thresholds based on the grayscale levels.

The fundamental approach of threshold entails initially setting a threshold, after which all pixels are dichotomized into two classes based on the relationship between their feature values and the established threshold. If a pixel’s feature value surpasses the threshold, it is labeled as the object class; otherwise, it’s categorized as the background. By judiciously choosing the threshold, one can effectively isolate the image’s subject from its surroundings. Given an original image denoted by f(x,y), a feature value T is identified within the image, leading to the segmentation of the original into two distinct components, forming the resulting segmented image.


(1) $$g(x,y)=\left\{ \begin{array}{l} b_{0},(fx,y)<t\\ b_{1},(fx,y)\geq t \end{array}\right.$$

If $b_{0}=0$ , $b_{1}=1$ , this refers to the image binarization process.

This paper utilizes threshold techniques for the automatic segmentation of various vegetation index images to extract pests and diseases regions. The following nine threshold methods are employed.

#### 3.3.1 OTSU

The Otsu (Kumar and Ramakrishnan, 2012) Threshold Technique, synonymous with the Maximum Variance Between Classes approach, stands as a prevalent and widely referenced algorithm within the threshold literature. Its core principle revolves around identifying the optimal gray-level value that effectively separates the foreground and background by maximizing the distinction between their respective classes. This process entails leveraging the image’s grayscale histogram to establish a suitable threshold, K, which partitions the image into two components: the foreground (Objective, O) and the background (Background, B). The algorithm optimizes for the highest between-class variance, as depicted in the equation below:


(2) $$e^{2}(K)=P_{0}{\left(\mu -\mu_{0}^{\prime }\right)}^{2}+P_{b}{\left(\mu -\mu_{b}^{\prime }\right)}^{2}$$

where μ signifies the average grayscale intensity of all pixels within the image, whereas $\mu_{0}^{\prime }$ and $\mu_{b}^{\prime }$ respectively indicate the mean grayscale intensities for the foreground (Objective) and background (Background) regions. The optimal threshold is established when the condition is fulfilled: K is selected in such a way that the between-class variance, denoted as $e^{2}(K)$ , is maximized.

#### 3.3.2 Block OTSU method

BOTSU (Liu and Wang, 2009) is carried out on a cell-by-cell basis for threshold segmentation, offering a more targeted and precise approach than applying threshold to the entire image. This method utilizes the OTSU algorithm, and its underlying principle is as follows: Let t denote the threshold value used to separate the foreground from the background. The proportion of foreground pixels within the image is $w_{0}$ , and their average grayscale intensity is $\mu_{0}$ . The proportion of background pixels is $w_{1}$ , and their average grayscale intensity is $\mu_{1}$ . The total average grayscale intensity of the image is:


(3) $$\mu =w_{0}\times \mu_{0}+w_{1}\times \mu_{1}$$

Beginning at the minimum grayscale value, iterate through all possible values of t until reaching the maximum. The optimal threshold is identified as t when the value $g=w_{0}\times {\left(\mu_{0}-\mu \right)}^{2}+w_{1}\times {\left(\mu_{1}-\mu \right)}^{2}$ is maximized. This expression represents the between-class variance. The two parts of the image separated by the threshold t, namely the foreground and background, make up the entire image. In this paper, the segmentation is executed on a 100x100 pixel region.

#### 3.3.3 Local dynamic threshold method

The LDT method (Venkatesh and Rosin, 1995) is mainly used in situations where the contrast is low and it is challenging to extract useful information using a global threshold. This method first partitions the image into distinct regions and calculates the segmentation threshold for each region, enabling adaptive computation of varying thresholds for different brightness levels in the image. Generally, the local dynamic threshold is determined based on the mean grayscale value and standard deviation within the neighborhood of the current pixel. For a grayscale image, if the coordinates of the current pixel are (x, y), and the neighborhood is centered at this point with a size of r×r, then g(x, y)denotes the grayscale value at position (x, y). The formula for calculating the mean grayscale value m(x, y) and grayscale variance s(x, y) within the r×r neighborhood is as follows:


(4) $$m(x,y)=\frac{1}{r^{2}}\sum_{i=x-\frac{r}{2}}^{x+\frac{r}{2}}\sum_{j=y-\frac{r}{2}}^{y+\frac{r}{2}}g(i,j)$$

(5) $$s(x,y)=\sqrt{\frac{1}{r^{2}}\sum_{i=x-\frac{r}{2}}^{x+\frac{r}{2}}\sum_{j=y-\frac{r}{2}}^{y+\frac{r}{2}}\left(g(i,j\right)-m(i,j){)}^{2}}$$

The local threshold T(x, y) at the pixel location (x, y) is determined by the mean and variance of the grayscale, and the formula for its calculation is provided below:


(6) $$T(x,y)=m(x,y)\cdot [1+k\cdot (\frac{s(x,y)}{R}-1)]$$

where R denotes the dynamic range of the standard deviation, which is commonly set to R = 128 for 8-bit grayscale images. The parameter k is a correction factor, generally fulfilling the condition 0< k< 1.

#### 3.3.4 Weller adaptive threshold

The WAT technique (Wellner, 1993) employs an adaptive pixel segmentation technique. The process involves iterating through each pixel in the image and calculating the moving average of the preceding contiguous set of pixels. For a given pixel, if its intensity is notably greater than this average, it is assigned a white value (1), otherwise, it’s marked as black (0). Let $p_{n}$ denotes the pixel value at position n in the image, the sum of the last s pixels at that location as $f_{s}(n)$ , and the resulting image T(n) as either 1 (white) or 0 (black), depending on whether it is darker than T% of the average of the previous s pixels. The formula can be expressed mathematically as follows:


(7) $$T(n)=\left\{ \begin{array}{l} \begin{array}{cc} 1 & if\;p_{n}<(\frac{f_{s}(n)}{s}) \end{array}(\frac{100-t}{100})\\ \begin{array}{cc} 0 & otherwise \end{array} \end{array}\right.$$

#### 3.3.5 Maximum entropy threshold method

The ME, as one of the frequently applied criteria in multi-threshold, employs the principle of entropy maximization to characterize the equivalence of conditions. The MET technique (Wong and Sahoo, 1989) segments the image into foreground and background entropy regions, and assesses the image information by optimizing the sum of the quantized entropy. Given a grayscale image with 256 levels of pixel grayscale, it is necessary to identify a set of thresholds $\left\{k_{1},k_{2},\ldots ,k_{n}\right\}(n>0)$ to segment the target image into n+1 parts, with each part corresponding to $\left\{C_{1},C_{2},\ldots ,C_{n}\right\}$ . The entropy values for each of these parts as determined by ME are as follows:


(8) $$\left\{ \begin{array}{l} C_{0}=\sum_{i=0}^{k_{1}}\frac{P_{i}}{U_{0}}\times \ln\frac{P_{i}}{U_{0}}\\ C_{1}=-\sum_{i=k_{1}+1}^{k_{2}}\frac{P_{i}}{U_{1}}\times \ln\frac{P_{i}}{U_{1}}\\ C_{n}=-\sum_{i=k_{n}+1}^{255}\frac{P_{i}}{U_{n}}\times \ln\frac{P_{i}}{U_{n}} \end{array}\right.$$

where $p_{i}$ represents the probability of the grayscale value of any pixel in the image relative to the average grayscale value of the region, and $U_{n}$ denote the cumulative probability of the occurrence of the (n+1)th subset. It can be mathematically expressed as:


(9) $$f(\{k_{1},k_{2},\ldots ,k_{n}\})=C_{0}+C_{1}+\cdots +C_{n}$$

#### 3.3.6 Double-peak method

The DPM (Prewitt and Mendelsohn, 1966) represents a straightforward and efficient image segmentation approach. Its core idea is to determine the segmentation threshold between the foreground and background by pinpointing the two peaks within the image’s grayscale histogram. Subsequently, the global threshold is established at the grayscale value that lies midway between these two peaks. Generally, the first peak, which aligns with the histogram’s maximum value, is labeled as p, while the second peak is calculated in accordance with the following equation:


(10) $$\arg\;\max\;D={\left(x-p\right)}^{2}*hist(x)$$

where x represents the grayscale value, with a range of 0 to 255, corresponding to the grayscale histogram’s values hits(x) . The value of x that results in the maximum value of D is equivalent to the grayscale value of the second peak. Subsequently, the threshold value is determined by choosing the smallest grayscale value that falls between the two peak values.

#### 3.3.7 Minimum error method

The grayscale image processed by the minimum error method (Kittler and Illingworth, 1986) is composed of two elements: the target and the background, both presumed to adhere to a Gaussian mixture distribution. By computing the mean and variance for both the target and the background, the objective function is derived for minimizing classification error, as expressed in following equation:


(11) $$E=\sum_{i=1}^{n}w_{i}\cdot \sigma_{i}^{2}$$

where n represents the number of gray levels, $w_{i}$ is the ratio of the number of pixels at the i-th grayscale level to the total number of pixels, and $\sigma_{i}^{2}$ represent the weighted variance between the target and background when using the i-th grayscale level as the threshold. The threshold that yields the smallest error is deemed the optimal threshold. Ultimately, this optimal threshold is applied to transform the image into a binary form.

#### 3.3.8 Iterative threshold method

The essence of the ITM technique (Ridler and Calvard, 1978) revolves around adaptively adjusting the threshold for automatic image segmentation. The sequence of actions involves: initially setting a threshold $T_{h}$ , dividing the image pixels into two classes - foreground and background, calculating the average grayscale value $m_{A}$ and $m_{B}$ for each, and then utilizing the arithmetic mean of these averages as the new threshold $T_{hnew}$ . The algorithm decides on termination by comparing the disparity between the current and previous iteration’s thresholds. If the difference falls below a predetermined limit, the iteration halts; otherwise, the procedure repeats. This continuous cycle persists until the most suitable segmentation threshold is attained.

#### 3.3.9 Combining global thresholds with local thresholds

Firstly, an initial global threshold T is established based on the average grayscale value of the image, leading to the division of pixels into two categories: Category G1, comprising pixels with grayscale values exceeding T, and Category G2, encompassing pixels with grayscale values below T. Subsequently, the mean grayscale values for G1 and G2, represented as $m_{1}$ and $m_{1}$ , are determined. The new threshold is then determined as the average of these mean values, denoted as $(m_{1}+m_{2})/2$ $(m_{1}+m_{2})/2$ . The image is binarized once more using this revised threshold. This procedure is carried out iteratively, with the algorithm proceeding until the difference between the global thresholds calculated in two successive iterations reaches zero. This iterative process refines the selection of the global threshold, thereby yielding a more precise binary segmentation (Yang et al., 2017). The computation of the mean thresholds for the two categories is as follows: $m_{1}=\frac{1}{\left|G_{1}\right|}\sum_{x\in G_{1}}x$ and $m_{2}=\frac{1}{\left|G_{2}\right|}\sum_{x\in G_{2}}x$ . The global threshold is then updated to this new value $T_{n+1}=\frac{m_{1}+m_{2}}{2}$ .

### 3.4 Random Forest variable importance

Random Forest is a popular ensemble learning technique extensively applied within the field of machine learning. It addresses a given problem by building numerous decision trees and arrives at the ultimate classification outcome through a voting mechanism. In contrast to alternative approaches, Random Forest stands out for its user-friendly nature, high robustness, and its ability to avoid overfitting.

Feature importance evaluation is used to calculate the importance of sample features, quantitatively describing the contribution of features to classification or regression. Random forests can be used to assess feature importance, which, from another perspective, is a built-in tool of random forests, mainly divided into two methods: (1) Mean Decrease Impurity (MDI), which measures the importance of a node by statistically calculating the decrease in impurity when the node is split; (2) Mean Decrease Accuracy (MDA), which involves randomly permuting the values of a feature in the out-of-bag (OOB) data set and then repredicting, calculating the importance of the feature by measuring the degree of decrease in classification/regression accuracy. MDI uses training set data and can directly obtain MDI feature importance assessment values after RF training is completed; MDA uses OOB data and requires running a separate feature importance evaluation program after RF training is completed. Since MDI uses training data exclusively, it may affect the accuracy of the assessment and is more inclined to increase the weight of high cardinality features. In comparison, the results of MDA are more accurate, which is used in this paper.

Feature selection plays a pivotal role in the Random Forest algorithm, involving the evaluation and ranking of the significance of various features. Let’s consider a dataset S that includes m samples and P feature variables, with y being the class label associated with each sample. The Random Forest algorithm constructs T decision trees through T iterations of bootstrap sampling. Due to this sampling approach, not all samples are used in the building of each tree; the ones that are not used are referred to as OOB samples. The OOB error e is determined by validating against the OOB samples, and this error is recalculated after randomly altering a particular feature. The degree of change in the OOB error, both before and after the feature modification, reflects the importance of that feature. The importance metric J for feature x can be formulated as follows:


(12) $$J_{a}(x_{j})=\frac{1}{T}\sum_{B_{k}\in S}\frac{1}{\left|B_{k}\right|}(\sum_{i\in B_{k}}I(h_{k}^{{\bar{x}}_{j}}(i)\neq y_{i}-I(h_{k}(i)\neq y_{i})))$$

where the predicted label output by the model is denoted by $h_{k}(i)$ , the genuine label from the validation set is represented by $y_{i}$ , and any changes to the feature $x_{j}$ lead to the modified prediction indicated by $h_{k}^{{\bar{x}}_{j}}(i)$ .

In this experiment, a 300 x 300 pixel area was chosen from a 1000 x 1000 pixel image to record the values of 27 indices. These 27 indices act as independent variables, while the classification outcomes of the ground truth map, denoted by 0 or 1, serve as the dependent variable. Utilizing the Random Forest algorithm, the importance score for each of these features was determined and subsequently the 27 features were ranked in order of decreasing importance.

### 3.5 Correlation coefficient iterative analysis

In dealing with the issue of multicollinearity, the CCIA provides an effective and intuitive solution. The core idea of this method is to identify and remove highly correlated feature pairs within the feature set. By iteratively selecting and eliminating variables with high correlation, it is possible to gradually construct a feature set with low multicollinearity, thereby enhancing the interpretability and predictive performance of the model.

The step-by-step iterative analysis can be detailed as follows: Initially, a correlation coefficient matrix for all features is generated to evaluate their linear associations. The matrix then highlights feature pairs with correlation coefficients exceeding a set threshold. For each pair of highly correlated features, one feature is chosen to keep while the other is eliminated to reduce collinearity. This iterative elimination continues until the correlation coefficients of all feature pairs fall below the threshold. After the removal of features with high correlation, the remaining ones are used to construct a multiple linear regression model that minimizes collinearity.

Mathematically, the correlation coefficient between feature i and feature j can be calculated using the following formula:


(13) $$\rho_{ij}=\frac{\sum \left(x_{ik}-{\bar{x}}_{i})(x_{jk}-{\bar{x}}_{j}\right)}{\sqrt{\sum {\left(x_{ik}-{\bar{x}}_{i}\right)}^{2}\sum {\left(x_{jk}-{\bar{x}}_{j}\right)}^{2}}}$$

where $x_{ik}$ and $x_{jk}$ signify the values of the i-th and j-th feature for the k-th data point, whereas ${\bar{x}}_{i}$ and ${\bar{x}}_{j}$ denote their corresponding mean values. The computation of correlation serves to manage the issue of multicollinearity among features effectively during feature selection. By using this method, it can be ensured that multicollinearity among features is effectively controlled during the feature selection process, thereby laying the foundation for constructing a robust regression model.

### 3.6 Beluga whale optimization

The BWO (Zhong et al., 2022) is inspired by the behavioral traits of beluga whales. It models the whales’ activities of swimming, foraging, and diving through a three-phase framework: exploration, exploitation, and whale diving. Beluga whales are operational as search agents, navigating the search space by adjusting their position vectors. During the exploration phase, random selection of whales ensures a comprehensive search across the scanning area. In the exploitation phase, the algorithm focuses on local search within the scanning space, utilizing the charged flight technique to improve convergence. Following the completion of exploration and exploitation in each iteration, the algorithm transitions to the whale diving stage of optimization.

Since the BWO algorithm is based on a population mechanism, beluga whales are regarded as search agents here. Each beluga whale acts as a candidate solution during the optimization process and is constantly updated. The position matrix of the search agents is modeled as follows:


(14) $$X=\left(\begin{array}{cccc} x_{1,1} & x_{1,2} & \cdots & x_{1,d}\\ x_{2,1} & x_{2,2} & \cdots & x_{2,d}\\ \vdots & \vdots & \ddots & \vdots \\ x_{n,1} & x_{n,2} & \cdots & x_{n,d} \end{array}\right)$$

where the number of beluga whales is denoted by n, and d represents the dimensionality of the problem variables. The fitness values corresponding to all beluga whales are stored as follows:


(15) $$F_{X}=\left(\begin{array}{c} f(x_{1,1},x_{1,2},\cdots ,x_{1,d}\\ f(x_{2,1},x_{2,2},\cdots ,x_{2,d}\\ \vdots \\ f(x_{n,1},x_{n,2},\cdots ,x_{n,d} \end{array}\right)$$

The BWO algorithm adjusts the transition from exploration to exploitation using the balance factor Bf, as defined by the equation:


(16) $$B_{f}=B_{0}\cdot (1-\frac{t}{2T})$$

where t represents the current iteration count, T denotes the total number of iterations, and $B_{0}$ is a random value between -5 and 5, which varies at each iteration. When the balance factor $B_{f}$ is greater than 0.5, this indicates an exploration phase, while a $B_{f}$ value less than or equal to 0.5 indicates a development phase.

(1) Exploration Phase

The exploration phase of the BWO algorithm is inspired by the swimming behavior of beluga whales. The position of the search agent is determined by the cooperative swimming of the beluga whales, and the position update of the beluga whales is as follows:


(17) $$\left\{ \begin{array}{l} x_{i,j}^{t+1}=x_{i,p}^{t}+x_{r,p}^{t}-x_{i,p}^{t})(1+r_{1})\times sin(2\pi r_{2}),\;j\;is\;even\\ x_{i,j}^{t+1}=x_{i,p}^{t}+x_{r,p}^{t}-x_{i,p}^{t})(1+r_{1})\times \cos(2\pi r_{2}),\;j\;is\;odd \end{array}\right.$$

where $x_{i,j}^{t+1}$ denotes the position of the i-th individual in the j-th dimension at the next iteration; if the problem dimension is assumed to be D, then p represents a random integer within the range [1, D], thus, $x_{i,j}^{t}$ denotes the position of the i-th individual in the randomly selected dimension p under the current iteration; r is also a random integer, where the population size is assumed to be N, and r is a random integer within the range [1, N], thus, $x_{r,p}^{t}$ denotes the position of the randomly selected individual r in the randomly selected dimension p under the current iteration; and r1 and r2 are both random numbers between (0, 1).

(2) Development Phase

The inspiration for the development phase of the BWO algorithm is derived from the feeding behavior of beluga whales. In order to improve the algorithm’s convergence, the Levy flight strategy is incorporated in the development phase, assuming that beluga whales utilize this flight strategy to capture prey. The mathematical model for this strategy is represented as


(18) $$x_{i}^{t+1}=r_{3}\cdot x_{best}^{t}-r_{4}\cdot x_{i}^{t}+C_{1}\cdot L_{F}\cdot (x_{r}^{t}-x_{i}^{t})$$

Where: $x_{i}^{t}$ and $x_{r}^{t}$ are the current positions of the i-th beluga whale and a random beluga whale, respectively; $x_{i}^{t+1}$ is the new position of the i-th beluga whale; $x_{best}^{t}$ is the best position of the beluga whales; $r_{3}$ and $r_{4}$ are random numbers between 0 and 1; $C_{1}=2\times r_{4}(1-\frac{t}{T})$ is the random jump intensity; and $L_{F}$ is a random number following the Levy distribution, which is calculated as follows:


(19) $$L_{F}=0.05\times \frac{\mu \cdot \sigma }{|V{|}^{\frac{1}{\beta }}}$$

(20) $$\sigma ={\left(\frac{\Gamma (1+\beta )\times sin(\pi \cdot \frac{\beta }{2})}{\Gamma (\frac{1+\beta }{2}\times \beta \times 2^{\frac{\beta -1}{2}}}\right)}^{\frac{1}{\beta }}$$

where both μ and V represent random numbers that follow a normal distribution, while β is a constant with a value of 1.5.

(3) Whale Falling Phase:

In order to maintain a constant population size, a position update formula is created by utilizing the current location of the beluga whale and the step size of the whale’s descent.


(21) $$x_{i}^{t+1}=r_{5}\cdot x_{i}^{t}-r_{6}\cdot x_{r}^{t}+r_{7}\cdot x_{step}$$

(22) $$x_{step}=\exp\frac{-C_{2}\cdot t}{T}\cdot (U_{b}-L_{b})$$

where $r_{5}$ , $r_{6}$ and $r_{7}$ are random numbers between (0, 1), $x_{step}$ denotes the step length of the whale’s fall, $C_{2}=2W_{f}\times n$ is a step factor related to the probability of the whale’s descent and the population size; $U_{b}$ and $L_{b}$ are the upper and lower bounds for the variable. It is evident that the step length of the whale’s descent is influenced by the problem variable boundaries, the current iteration, and the maximum iteration count.

The probability of the whale falling is designed as a linear function.


(23) $$W_{f}=0.1-0.05\frac{t}{T}$$

More detailed implementation information could be found in Ref (Zhong et al., 2022).

### 3.7 BWO-weighted new vegetation index

After RFVI or CCIA, a certain number of vegetation indices are selected as input for the subsequent BWO. Assuming the selected vegetation indices are denoted as $<VI_{1},VI_{2},\ldots ,VI_{n}>$ , training region images and their corresponding ground truth value regions are chosen, and the BWO algorithm is applied to the n vegetation indices with weighting. For this experiment, the value of *n* can be 5, 10, 15, 20, or 27. The population size is set to PS, and the initial position of each whale is set to $<w_{1},w_{2},\ldots ,w_{n}>$ . The new vegetation index is then defined as:


(24) $$VI\_new=\sum_{i=1}^{n}w_{i}\times VI_{i}$$

After acquiring PS new vegetation indices, a specific threshold segmentation algorithm, for instance, OTSU, is chosen to segment these indices. The outcomes of this segmentation are then matched against the corresponding ground truth images to compute the accuracy assessment metric CSI of training area, generating PS CSI values. The CSI values act as the fitness measure, guiding the beluga whales’ behavior to shift between exploration and exploitation modes based on the balance factor *B_f_*. The whales’ actions are fine-tuned, and the optimal candidate is selected based on the descent probability *W_f_*. This process is iterated until the set number of cycles is completed, pinpointing the most suitable candidate as the definitive weight. This weight is then applied to the aggregate images of the n vegetation indices to derive the final new vegetation index, following the formula for the new index. It’s crucial to note that different threshold segmentation algorithms will result in different weights and new vegetation indices, providing essential variables for subsequent ensemble learning methods.

Since it is not possible to determine in advance how many variables to choose for better suitability, following the approach of most studies, multiple variable combinations are selected to analyze the impact of the number of variables on accuracy. Specifically, this paper sets the number of beluga whales to 50, with the dimensionality of the individual whale position vector being 5, 10, 15, 20, and 27, corresponding to the number of selected vegetation index variables. The search space range is from -10 to 10, and the number of iterations is fixed at 50. Following this, nine threshold segmentation techniques are utilized to refine the BWO of the n new vegetation indices, which are then integrated to facilitate the precise monitoring of trees affected by pests and diseases.

### 3.8 BWO-weighted ensemble learning strategy

Ensemble learning involves training multiple fundamental classifiers and combining them either sequentially or in parallel. This is followed by the application of a particular ensemble strategy to accomplish the learning task, with the goal of minimizing variance, bias, and enhancing prediction accuracy. The main challenge in ensemble learning is the choice of the ensemble strategy, with common approaches being averaging, voting, and learning methods. Averaging is appropriate for numerical regression prediction issues, whereas voting and learning methods are frequently applied to classification tasks. Voting, a strategy often employed in ensemble learning recognition tasks, adheres to the “majority rules” principle. It encompasses absolute majority voting, relative majority voting, and weighted voting. For a given task, an integrated model $F(x_{i})$ is constructed based on the voting mechanism, with the assumption that the ensemble consists of M basic classification outcomes $\left\{f_{1},f_{2},\ldots f_{M}\right\}$ . The final classification category for each pixel is determined by selecting the category that receives the most votes, which constitutes the “majority s voting” strategy.

Within the scope of this paper, the fundamental classification outcomes are the detection images derived from the threshold segmentation technique. Given the varied performance of these fundamental classification outcomes, this paper proposes a BWO-weighted voting approach. By utilizing BWO to assign weights to each fundamental classification outcome and optimizing these weights through a fitness function, the ultimate voting result is ascertained by employing a majority voting strategy.

Here are the detailed steps: Nine distinct threshold segmentation techniques are applied to the new vegetation index, generating nine foundational classification results. The nine base classifications from the sub-region employed in the creation of the new vegetation index were taken as inputs for the BWO algorithm, along with parameters like the balance factor $B_{f}$ , fall probability $W_{f}$ , population size, and iteration count. The fitness function is defined by the CSI values. With each iteration, the weights for the base classifications are adjusted based on the CSI values. Unlike traditional BWO, the weights in this study are integers ranging from 0 to 100. For each beluga whale, a number of copies of each base classification equal to its weight are made; for example, a classification with a weight of 50 is replicated 50 times. After all classifications are duplicated, a comprehensive set $\sum_{I=1}^{9}w_{i}$ including all classifications is formed, and a majority voting mechanism is applied to each pixel within this set to produce the final ensemble outcome. The CSI value between this ensemble outcome and the actual reference data is calculated to assess the fitness of the individual. Depending on the balance factor, the algorithm advances to either an exploration or exploitation phase, experiences the fall process, and ends after the maximum number of iterations is reached. The best individual and its corresponding weights are then chosen as the final weights. These final weights are then applied to the large-scale image made up of the base classifications, and the ultimate pest and disease detection result is obtained through majority voting for each pixel.

## 4 Experimental results and analysis

### 4.1 Evaluation metrics

In the assessment of classification outcomes, the prevalent evaluation technique is the confusion matrix (Friedl et al., 2010), often referred to as the Error Matrix based on Sample. It is derived from the counts of correctly classified pixels per class, misclassified pixels to other classes, and undetected instances. This matrix functions as a benchmark to evaluate the precision of pixel classification against actual ground-truth categories. This study employs the confusion matrix for validation and performance evaluation, with a thorough examination of errors in pests and diseases classification carried out through metrics such as: Probability of Detection (POD), False Alarm Rate (FAR), CSI, Overall Accuracy (OA), Kappa Coefficient. Given that the pests and diseases detection in the present investigation is a binary classification, the corresponding confusion matrix is presented in **Table 2**.

**Table 2:** The confusion matrix.

	Predicted as Positive	Predicted as Negative
Labeled as Positive	True Positive (TP)	False Negative (FN)
Labeled as Negative	False Positive (FP)	True Negative (TN)

(1) CSI, also known as Accuracy, is a measure used to evaluate the precision of identifying positive cases. CSI denotes the ratio of correctly classified positive cases to all positive cases:


(25) $$CSI=\frac{TP}{TP+FN+FP}$$

(2) POD, alternatively known as Sensitivity or True Positive Rate (TPR), evaluates the capability to accurately recognize positive cases. POD signifies the ratio of samples accurately classified as positive within the total population of actual positive samples:


(26) $$POD=\frac{TP}{TP+FN}$$

(3) FAR, also known as the Miss Rate or False Positive Rate (FPR), is a metric used to evaluate the error rate in identifying negative instances. It indicates the percentage of negative samples that are incorrectly labeled as positive relative to the total number of actual negative samples:


(27) $$FAR=\frac{FP}{FP+TN}$$

(4) The OA serves as a quantitative measure representing the extent to which every individual sample is accurately assigned to its corresponding category:


(28) $$OA=\frac{TP+TN}{TP+FN+FP+TN}$$

(5) The Kappa coefficient is a metric of the accuracy of the overall image classification result, with a maximum value of 1. A larger Kappa coefficient indicates a greater degree of consistency between the classified results and the ground truth.


(29) $$Kappa=\frac{P_{0}+P_{c}}{1-P_{c}}$$

where,


(30) P=OA

(31) $$P_{c}=\frac{\left(TP+FN)(TP+FP)+(FN+TN)(FP+TN\right)}{{\left(TP+FN+FP+TN\right)}^{2}}$$

### 4.2 Correlation analysis between indices

**Figure 6:**
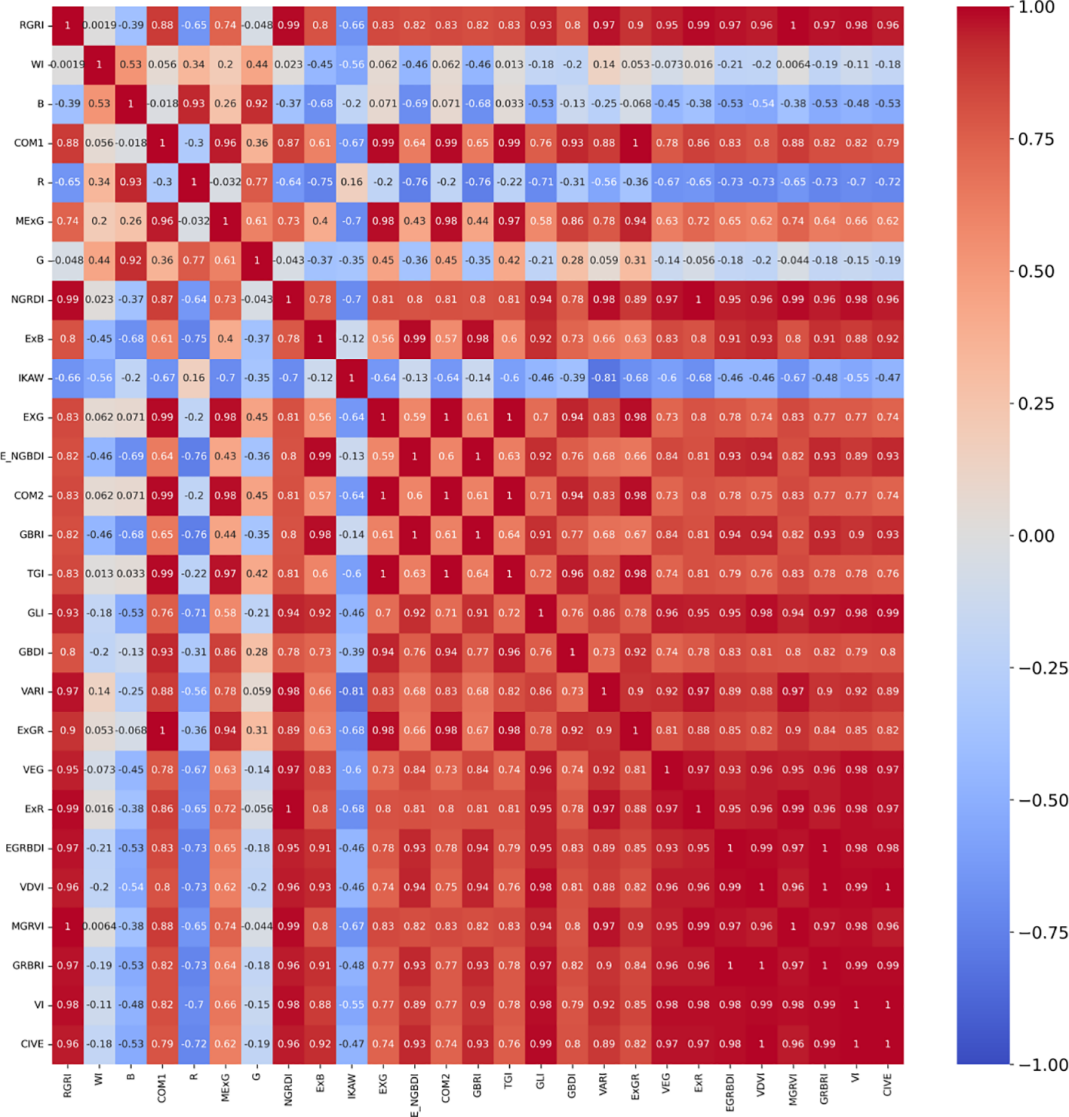
Heatmaps of correlation coefficients for the 27 features.

The correlation analysis is a quantitative technique that investigates the interdependence of data, evaluates the multicollinearity among indices, and furnishes a theoretical foundation for feature extraction. This paper computed the correlation coefficients for the 27 normalized attributes (comprising 24 vegetation indices and the three RGB bands), resulting in heatmaps displayed in **Figure 6** for visual representation of these relationships.

**Figure 7:**
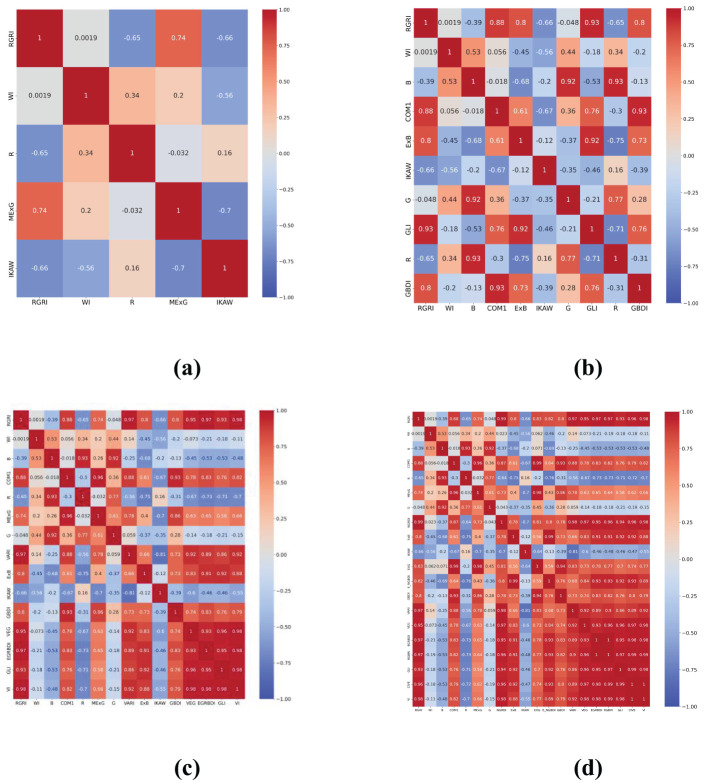
Heatmaps of Correlation Coefficients for the 5, 10, 15 and 20 Features **(A)** 5 features; **(B)** 10 features; **(C)** 15 features; **(D)** 20 features.

The red color in the heatmap indicates a higher positive correlation, while the blue color indicates a higher negative correlation, and green represents a lower correlation. 5, 10, 15, and 20 features are selected for further iterative optimization, and the generated heatmap are shown in **Figure 7**.

### 4.3 Feature importance assessment

**Figure 8:**
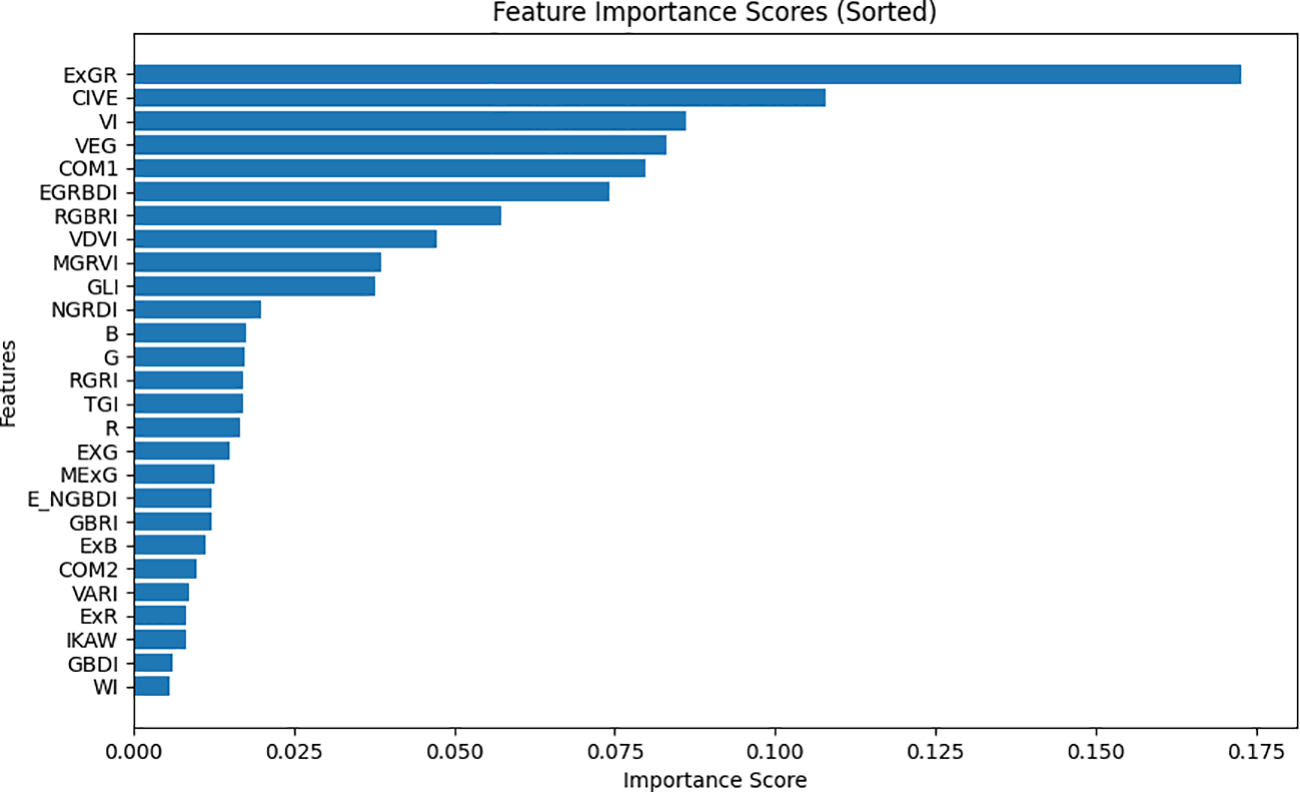
The sorting results of random forest importance.

To assess the performance of 27 variables (comprising 24 vegetation indices and the R, G, B bands) in monitoring pests and diseases of CCB, a dataset was assembled from 300x300 sub-regions within the study area. Employing a random forest model, the relative significance of these 27 variables was determined. Initially, 27 distinct feature images were generated for this small region as independent variable, with the disease map serving as the dependent variable, represented by binary values (0 and 1). The random forest algorithm was employed to calculate the importance score for each variable, and the features were subsequently arranged in descending order of their importance. The results are illustrated in **Figure 8**.

According to **Figure 8**, ExGR and COM1 are the top two important features, considerably surpassing the other indices, which implies that these two features are more crucial for classification. Close behind are CIVE and VEG, with eight other indices, while B, G, and 15 other indices have relatively low importance scores, indicating their limited influence on accurate classification.

### 4.4 The validity of BWO-weighted new vegetation index using single OTSU

To verify the efficacy of the proposed BWO-weighted new vegetation index, the RFVI was utilized to select the top 10 most important indices and the CCIA was used to choose the 10 least correlated indices. By employing the OTSU algorithm, binary classification maps were generated for these 10 indices, representing pests and diseases detection. The BWO was then applied to adaptively weight these 10 features, and the resulting new vegetation index images were segmented using OTSU adaptively to produce the final pests and diseases detection map.

**Figure 9:**
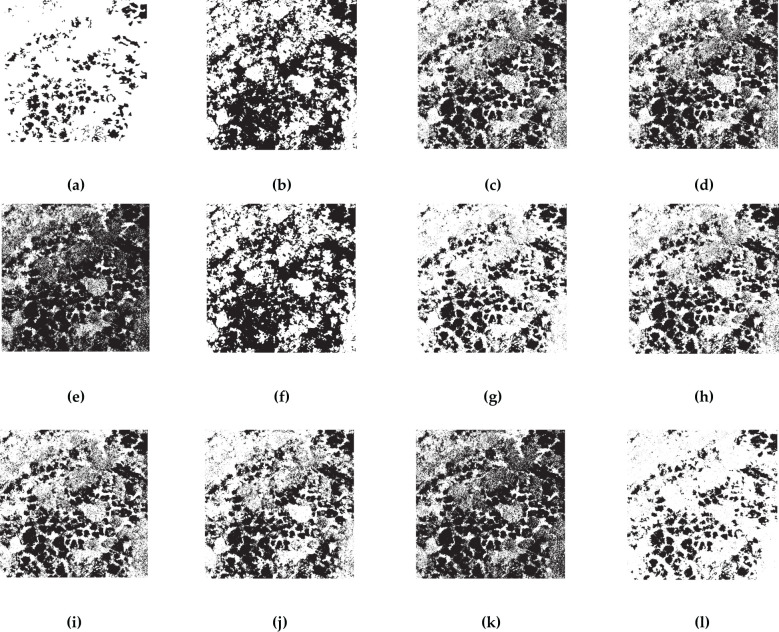
OTSU results for 10 indices selected by RFVI. The black areas indicate CCB trees with pest and disease, while the white areas indicate healthy trees. **(A)** ground truth; **(B)** ExGR; **(C)** CIVE; **(D)** VI; **(E)** VEG; **(F)** COM1; **(G)** EGRBDI; **(H)** RGBRI; **(I)** VDVI; **(J)** MGRVI; **(K)** GLI; **(L)** BWO-weighted new vegetation index.

**Figure 10:**
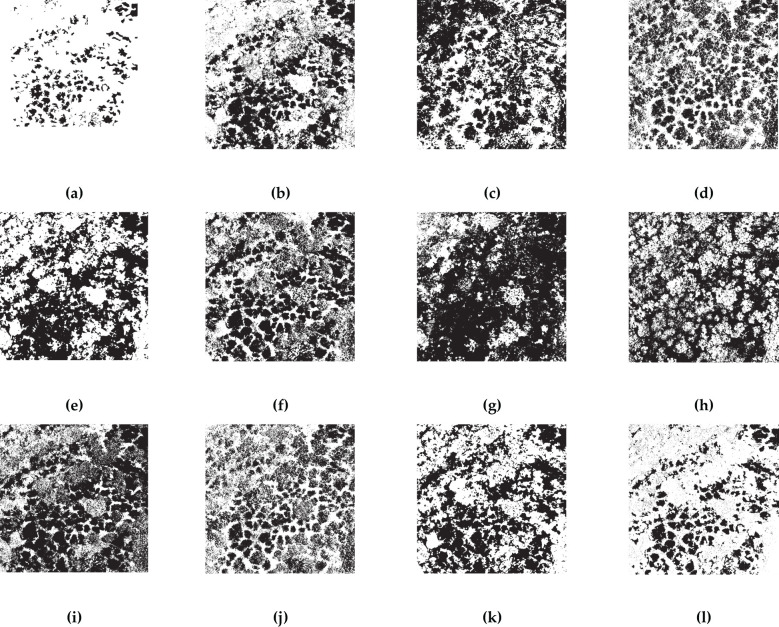
OTSU results for 10 indices selected by CCIA. The black areas indicate CCB trees with pest and disease, while the white areas indicate healthy trees. **(A)** ground truth; **(B)** RGRI; **(C)** WI; **(D)** B; **(E)** COM1; **(F)** ExB; **(G)** IKAW; **(H)** G; **(I)** GLI; **(J)** R; **(K)** GBDI; **(L)** BWO-weighted new vegetation index.

**Figures 9**, **10** illustrate the OTSU threshold segmentation outcomes for the sets of 10 indices chosen by RFVI and CCIA, respectively. The black areas in these figures represent detected pests and diseases plants. It is apparent that, when compared to the ground truth, the direct segmentation of the original vegetation index yields unsatisfactory results, with significant misclassification and false negatives. Conversely, the segmentation method using the 10 weighted indices through BWO (i.e. BWO-weighted new vegetation index) surpasses the single vegetation index threshold, as demonstrated by the substantial reduction in false negatives and a closer distribution and positioning of pests and diseases trees, as shown in **Figures 9L**, **10L**.

**Tables 3**, **4** display the calculated accuracy metrics for the detection results. The direct OTSU segmentation of the indices is not satisfactory, as shown by a low CSI score, elevated POD and FAR values, indicating a propensity for over-segmenting pests and diseases pixels, signified by a low Kappa coefficient. The OTSU threshold segmentation of BWO-weighted new vegetation index outperforms the single vegetation index approach across all evaluation metrics, demonstrating that BWO successfully amalgamates valuable information from each index, generating new index capable of distinguishing pests and diseases areas from healthy ones. The distinctiveness of the indices is crucial, as each index captures a unique attribute, leading to numerous false positives in single-index OTSU segmentation. The results also underscore the advantages of BWO-weighted new vegetation index segmentation. The RFVI-selected indices exhibit higher precision in both OTSU segmentation and BWO-weighted new vegetation index processing compared to the ten chosen by the CCIA, suggesting that RFVI better reflects the relative performance among multiple indices.

**Table 3:** The classification accuracy of the OTSU segmentation applied to RFVI-selected single indices and BWO-weighted new vegetation index.

	CSI	POD	FAR	OA	Kappa
ExGR	0.3466	0.9932	0.6526	0.6599	0.3270
CIVE	0.3378	0.9858	0.6605	0.6498	0.3142
VI	0.3330	0.9876	0.6656	0.6414	0.3058
VEG	0.2461	0.9961	0.7537	0.4502	0.1467
COM1	0.3372	0.9937	0.6621	0.6455	0.3117
EGRBDI	0.5149	0.9553	0.4724	0.8350	0.5685
RGBRI	0.4166	0.9746	0.5788	0.7513	0.4368
VDVI	0.3683	0.9811	0.6291	0.6944	0.3638
MGRVI	0.3946	0.9791	0.6021	0.7263	0.4032
GLI	0.2712	0.9927	0.7282	0.5187	0.1959
BWO	0.6365	0.8777	0.3015	0.9029	0.6952

**Table 4:** The classification accuracy of the OTSU segmentation applied to CCIA-selected single indices and BWO-weighted new vegetation index.

	CSI	POD	FAR	OA	Kappa
RGRI	0.4045	0.9772	0.5917	0.7376	0.4180
WI	0.1930	0.6607	0.7858	0.5100	0.0788
B	0.2167	0.6419	0.7535	0.5893	0.1362
COM1	0.3372	0.9937	0.6621	0.6455	0.3117
ExB	0.3014	0.9770	0.6964	0.5916	0.2542
IKAW	0.2339	0.9915	0.7656	0.4152	0.1223
G	0.1962	0.7044	0.7861	0.4817	0.0686
GLI	0.2712	0.9927	0.7282	0.5187	0.1959
R	0.2710	0.7684	0.7050	0.6315	0.2253
GBDI	0.3656	0.9896	0.6330	0.6877	0.3578
BWO	0.5923	0.9087	0.3702	0.8844	0.6617

### 4.5 The validity of BWO-weighted new vegetation index using multiple threshold-based segment methods

In order to evaluate the detection accuracy of disease trees of multiple threshold segmentation algorithms under multiple indices conditions, based on the 10 indices selected respectively by RFVI and CCIA method in this paper, 9 threshold segmentation methods were used for automatic segmentation to obtain the detection results of pests and diseases trees for each index under each threshold segmentation method. Then for each threshold segmentation method, the average value of the detection accuracy of the 10 indices under this method was calculated as the average detection accuracy of this threshold segmentation method. At the same time, based on the 10 indices, the detection accuracy of the BWO-weighted new vegetation index by each threshold segmentation method was calculated.

**Figure 11:**
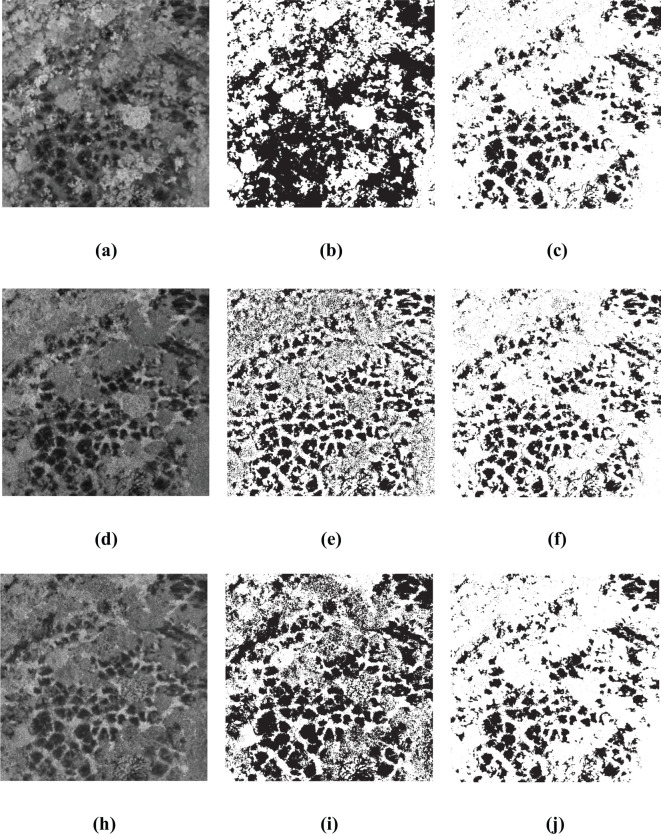
Some multiple threshold-based segment results for 10 indices selected by RFVI. The black areas indicate CCB trees with pest and disease, while the white areas indicate healthy trees. **(A)** ExGR; **(B)** OTSU for ExGR; **(C)** OTSU for BWO-weighted new vegetation index; **(D)** CIVE; **(E)** WAT for CIVE; **(F)** WAT for BWO-weighted new vegetation index; **(G)** ExB; **(H)** DPM for ExB; **(I)** DPM for BWO-weighted new vegetation index.

**Figure 12:**
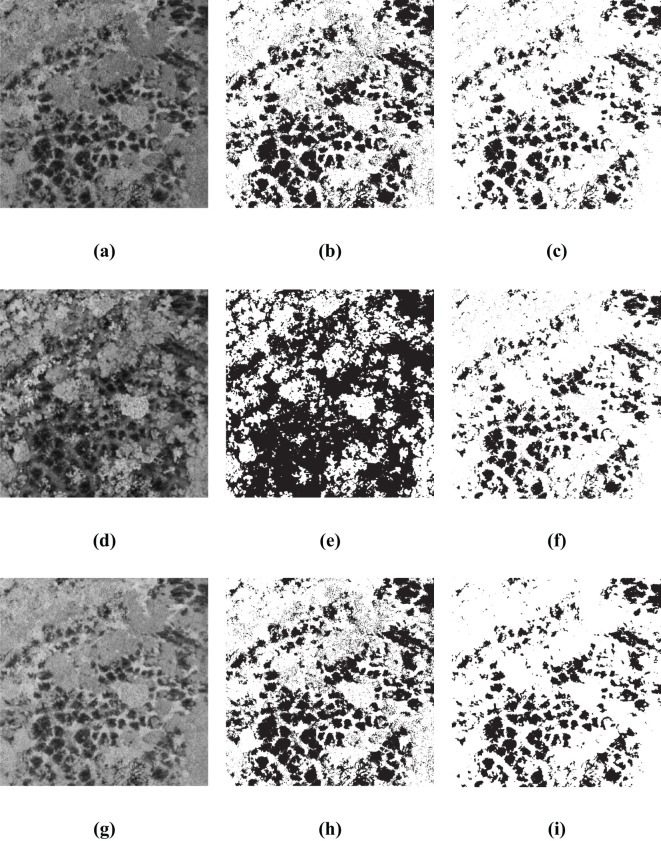
Some multiple threshold-based segment results for 10 indices selected by CCIA. The black areas indicate CCB trees with pest and disease, while the white areas indicate healthy trees. **(A)** RGBRI; **(B)** MET for RGBRI; **(C)** MET for BWO-weighted new vegetation index; **(D)** TGI; **(E)** ITM for TGI; **(F)** ITM for BWO-weighted new vegetation index; **(G)** EGRBDI; **(H)** CGLT for EGRBDI; **(I)** CGLT for BWO-weighted new vegetation index.

The **Figures 11**, **12** present the results of single-index and single-threshold segmentation for individual indices, as well as the segmentation outcomes of the BWO-weighted new vegetation index. The individual indices are chosen from the top 10 indices derived from RFVI and CCIA. From the figures, it can be observed that, irrespective of whether the indices are selected by RFVI or CCIA method, using any single-threshold segmentation method will result in severe noise and false negatives, primarily manifested in an excessive number of black areas (i.e., the pests and diseases trees) and a significant difference from the ground truth. However, the BWO-weighted new vegetation index, regardless of the single-threshold segmentation method used, can effectively segment the pests and diseases trees with a position similar to the ground truth, although there is still some noise, but its intensity is relatively low.

The quantitative evaluation results are shown in **Tables 5**, **6** as follows. The average detection accuracy of 9 threshold segmentation methods on 10 indices selected by the RFVI and the CCIA was calculated and expressed as “Th_Avg”, as well as the detection results on the BWO-weighted new vegetation index, represented as “Th_BWO”. It can be seen from the table that it is consistent with the subjective evaluation results in **Figures 11**, **12**. For the 10 indices selected by the RFVI, the average accuracy of each threshold method on all indices is relatively similar. The CSI index value is distributed around 0.30. The lowest is the LDT method, only 0.2579, and the highest is WAT, reaching 0.3974. Except for WAT, the POD values of all other methods exceed 0.95 and even reach 0.99, while the FAR also exceeds 0.6, indicating that there are a large number of false alarms in the detection results. The Th_BWO results are significantly better than Th_Avg, the CSI value exceeds 0.55, the OA exceeds 0.85, and the FAR remains at about 0.3, indicating that there are still certain false alarms, but the degree is much lighter than Th_Avg. For the 10 indices selected by the CCIA method, the overall trend is similar to the indices selected by the RFVI, but on each accuracy metric, the indices selected by the CCIA method are worse than those selected by the RFVI.

**Table 5:** The classification accuracy for multiple threshold segmentation methods based on the 10 indices selected by RFVI and the BWO-weighted new vegetation index.

	CSI		OA		Kappa		POD		FAR	
	Th_Avg	Th_BWO	Th_Avg	Th_BWO	Th_Avg	Th_BWO	Th_Avg	Th_BWO	Th_Avg	Th_BWO
OTSU	0.3566	0.6365	0.6572	0.9091	0.3374	0.7136	0.9839	0.8777	0.6405	0.3015
BOTSU	0.3079	0.5607	0.5962	0.8875	0.2662	0.6427	0.9502	0.8028	0.6858	0.3497
LDT	0.2579	0.5511	0.4362	0.8723	0.1620	0.6189	0.9500	0.8552	0.7156	0.3922
WAT	0.3974	0.5778	0.7677	0.8892	0.4322	0.6612	0.8597	0.8562	0.5674	0.3601
MET	0.2955	0.6444	0.4562	0.9120	0.2118	0.7215	0.9878	0.8797	0.7013	0.2933
DPM	0.3453	0.6277	0.6568	0.9071	0.3248	0.7065	0.9867	0.8665	0.6530	0.3051
MEM	0.3443	0.6525	0.6390	0.9171	0.3177	0.7315	0.9856	0.8618	0.6534	0.2713
ITM	0.2625	0.6522	0.4408	0.9166	0.1688	0.7308	0.9938	0.8653	0.7365	0.2741
CGLT	0.2785	0.6557	0.4791	0.9151	0.1956	0.7305	0.9939	0.886	0.7204	0.2838

**Table 6:** The classification accuracy for multiple threshold segmentation methods based on the 10 indices selected by CCIA and the BWO-weighted new vegetation index.

	CSI		OA		Kappa		POD		FAR	
	Th_Avg	Th_BWO	Th_Avg	Th_BWO	Th_Avg	Th_BWO	Th_Avg	Th_BWO	Th_Avg	Th_BWO
OTSU	0.2791	0.5923	0.5809	0.8856	0.2169	0.6631	0.8691	0.9087	0.7107	0.3702
BOTSU	0.2627	0.3528	0.5884	0.7196	0.1967	0.3580	0.7869	0.8535	0.7182	0.6245
LDT	0.1852	0.6013	0.4995	0.9078	0.0819	0.6844	0.7150	0.7662	0.7722	0.2635
WAT	0.2569	0.5416	0.7192	0.8735	0.2326	0.6229	0.5893	0.8463	0.6277	0.3993
MET	0.2473	0.6424	0.4645	0.9139	0.1538	0.7225	0.8746	0.8580	0.7384	0.2811
DPM	0.2792	0.6308	0.6028	0.9068	0.2211	0.7079	0.8321	0.8782	0.7060	0.3087
MEM	0.2675	0.6494	0.5824	0.9168	0.2045	0.7290	0.8268	0.8524	0.7136	0.2683
ITM	0.2255	0.6524	0.4548	0.9183	0.1232	0.7324	0.8628	0.8505	0.7579	0.2631
CGLT	0.2437	0.6592	0.4849	0.9168	0.1553	0.7347	0.8596	0.8831	0.7362	0.2778

### 4.6 The influence of the number of features on the detection results

**Figure 13:**
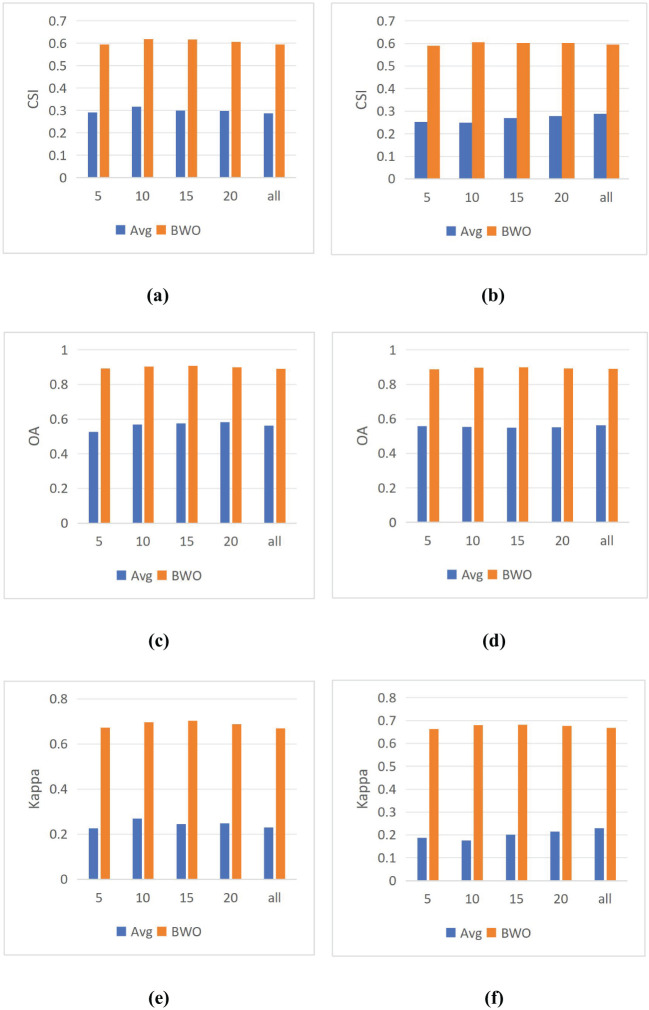
The influence of the number of features on the detection results. **(A)** CSI of RFVI **(B)** CSI of CCIA **(C)** OA of RVFI **(D)** OA of CCIA **(E)** Kappa of RFVI **(F)** Kappa of CCIA.

In the previous experiments, 10 indices were selected for threshold segmentation and BWO-weighted new vegetation index respectively by using RFVI and CCIA method. How will the selection of different numbers of indices affect the detection results? To solve this problem, in this section, the number of indices was set to 5, 10, 15, 20, and all features, and 9 threshold segmentation methods were used respectively to calculate the average value of the detection accuracy in each case of the number of indices, denoted as “Avg”. Then, in the same case of the number of indices, BWO-weighted new vegetation index was calculated, and 9 threshold segmentation methods were used to calculate the average value of the detection accuracy of the new index, denoted as “BWO”. The results are shown in **Figure 13** below:

The above figure shows that for both RFVI and CCIA methods, the number of indices has some impact, but it is relatively stable. In terms of trends, for “BWO”, the highest value is reached when there are 10 indices for both RFVI and CCIA methods, and the value decreases as the number of indices increases. This indicates that on this dataset, 10 indices already contain information that can better reflect the characteristics of pests and diseases, and more indices may introduce some redundancy and conflict, leading to a decrease in detection accuracy. For “Avg”, with the increase in the number of indices based on RFVI, the detection accuracy first increases and then decreases, while for the indices based on CCIA method, the detection accuracy increases with the number of indices. However, whether the trend is increasing or decreasing, the detection accuracy of “Avg” always remains at a relatively low level, far inferior to “BWO”, which also demonstrates the significant advantage of BWO.

### 4.7 The impact of the ensemble voting strategy

**Figure 14:**
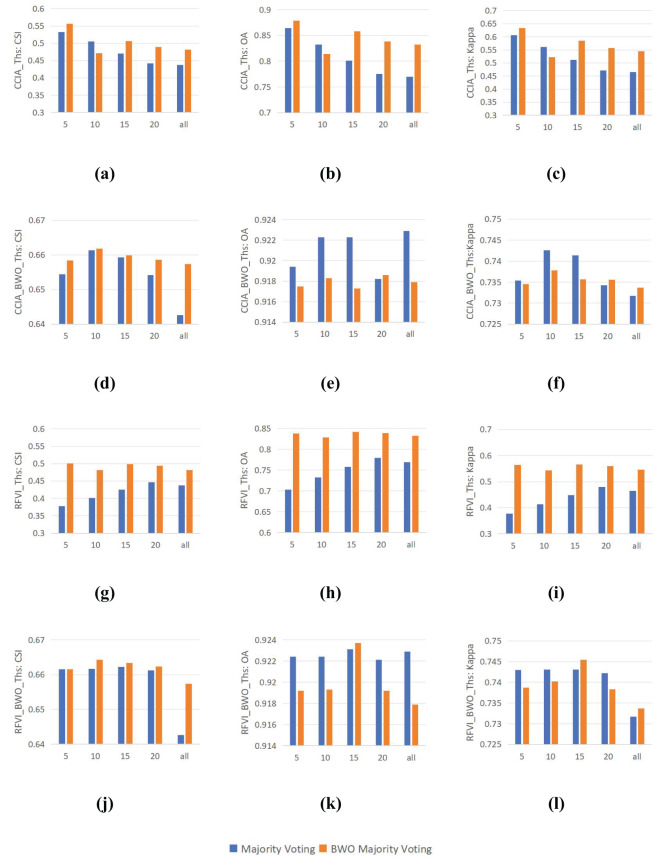
Impact of the Integrated Voting Strategy. **(A)** CSI of CCIA_Ths; **(B)** OA of CCIA_Ths; **(C)** Kappa of CCIA_Ths; **(D)** CSI of CCIA_BWO_Ths; **(E)** OA of CCIA_BWO_Ths; **(F)** Kappa of CCIA_BWO_Ths; **(G)** CSI of RFVI_Ths; **(H)** OA of RFVI_Ths; **(I)** Kappa of RFVI_Ths; **(J)** CSI of RFVI_BWO_Ths; **(K)** OA of RFVI_BWO_Ths; **(L)** Kappa of RFVI_BWO_Ths.

Ensemble learning is capable of synthesizing information from various components to achieve better results. In previous experiments, this paper has demonstrated that the BWO-weighted new vegetation index can significantly outperform a single index in terms of detection accuracy when applied to any adaptive threshold segmentation method. In this experiment, the performance differences between the conventional majority voting strategy and the BWO weighted voting strategy are examined. For multiple indices selected through RFVI and CCIA, each index is first segmented using nine threshold segmentation methods, resulting in a series of segmentation outcomes. For example, 10 indices yield 90 segmentation results, denoted with the suffix “_Ths”. These segmentation results are then ensembled using the majority voting strategy and the BWO weighted voting ensemble strategy to obtain the final detection results. As for the BWO-weighted new vegetation index, for a certain number of indices, a new vegetation index is obtained, which is segmented using nine threshold methods, resulting in nine segmentation outcomes, denoted with the suffix “_BWO_Ths”. These are then ensembled using the majority voting strategy and the BWO weighted voting ensemble strategy to achieve the final detection results. The accuracy of various strategies is evaluated using CSI, OA, and Kappa, with the results shown in **Figure 14** below:

It can be seen from the figure that, on the whole, whether it is CCIA or RFVI, the BWO weighted majority voting strategies (d)-(e) and (j)-(l) can have higher accuracy than the direct majority voting strategies (a)-(c) and (g)-(i) in various numbers of indices. Different numbers of indices have a significant impact on the final accuracy. For CCIA and RFVI, the highest accuracy of direct index segmentation occurs in the first 5 indices, while the highest accuracy of segmentation using the BWO-weighted new vegetation index occurs in the first 10 indices, meaning that selecting a few indices with the least correlation or the greatest importance often achieves higher detection accuracy than using all indices. By comparing (a) and (d), or (g) and (j), it can be seen that using the BWO-weighted new vegetation index can achieve significantly higher accuracy than the direct segmentation of indices, indicating the obvious advantage of the BWO-weighted new vegetation index proposed in this paper. In terms of the ensemble strategy, the BWO weighted majority voting strategy is superior to the direct majority voting strategy in most index selection methods and quantities, showing certain advantages. It should be pointed out that the optimization goal of BWO in this paper is the CSI index. The definition of the CSI index does not concern the situation where both the true value and the predicted value are TN, while OA and Kappa do consider it. When CSI reaches the maximum, it is also possible that OA and Kappa are not optimal. Therefore, there are situations where the values of direct majority voting in figures (e)(f)(k)(l) are higher than those of BWO weighted majority voting. However, in general, the three accuracy indicators still show a high degree of consistency.

### 4.8 More application scenarios

**Figure 15:**
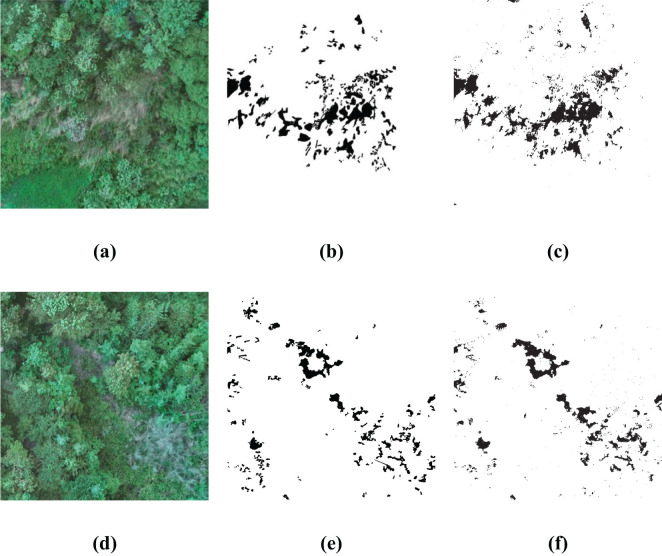
Generalization Capability in More Application Scenarios. **(A, D)** are original RGB images, **(B, E)** are the ground truth, **(C, F)** are the detection results.

In order to substantiate the effectiveness and generalizability of the approach outlined within this paper, a rigorous test were conducted by deploying the BWO-weighted newly formulated vegetation index and the BWO-weighted ensemble strategy methods. These methods were applied to a set of 10 indices derived from the RFVI, which had been previously trained in the earlier section of our paper. The purpose of this exercise was to assess how well the previously proposed method would perform when introduced to new and unseen scenarios. The outcomes of this test, which provide a visual representation of the method’s adaptability and accuracy, are depicted in **Figure 15**. This figure presents a comprehensive view of the results, allowing for a clear evaluation of the proposed method’s ability to generalize across different settings and conditions. Among them, (a) and (d) are the original RGB images, which show some CCB trees withered due to pests and diseases. (b) and (e) are the fine position ground truth maps of pests and diseases trees manually marked out, and (c) and (f) are the detection results of the method in this paper. It can be seen that, overall, the detection results have a very high similarity with the ground truth maps, especially the details of the pests and diseases trees. Although there is still some noise, the pests and diseases trees can basically be accurately detected.

The five accuracy metrics of CSI, POD, FAR, OA, and Kappa of the two scenarios were calculated based on the confusion matrix, and the results are shown in **Table 7** below:

**Table 7:** The accuracy metrics for more scenarios.

	CSI	POD	FAR	OA	Kappa
Scenario 1	0.5779	0.7233	0.2580	0.9594	0.7105
Scenario 2	0.5964	0.6901	0.1855	0.9759	0.7346

Comprehensive analysis of the data of two different scenarios reveals that the model trained by the previous dataset shows a high-precision recognition ability in other scenarios, with little difference in accuracy from the previous experiments, and can effectively identify the pests and diseases areas in RGB images. It is worth noting that the OA index not only assesses the recognition accuracy of the pests and diseases areas but also considers the non-pests and diseases areas, and the latter usually has a wider range. Therefore, the value of the OA index is very high. Overall, despite the challenges of new scenarios, the model still performs well, showing good generalization ability.

## 5 Conclusions

Pests and diseases monitoring of CCB trees is an important means to enhance its medicinal value. In response to the high cost and data processing difficulties associated with existing hyperspectral/multispectral sensors, this paper proposes a high-precision pests and diseases monitoring method based on visible light RGB images captured by UAV. Firstly, based on 24 RGB-derived vegetation indices, this paper introduces a new vegetation index based on the BWO algorithm. This new index is capable of integrating the advantageous features of multiple vegetation indices from various dimensions, forming a more comprehensive representation of the plant’s health status. Even when using the simplest threshold segmentation method, it can effectively detect pests and diseases-affected trees. Then, based on nine threshold segmentation methods, a new ensemble learning strategy based on BWO is proposed. By adaptively weighting the results of multiple threshold segmentation methods, it can stably achieve a better detection accuracy than a single threshold segmentation method. There may be collinearity among different vegetation indices, indicating a certain degree of information redundancy. This paper explores collinearity through the methods of RFVI and CCIA, showing that using a small number of vegetation indices can also achieve similar or even better detection accuracy. Real-world experimental results from a CCB planting base demonstrate that the proposed method can effectively detect pests and diseases-affected trees, which has certain value for the precise management of CCB. Deep learning, which has exhibited considerable potential and effectiveness in forest resource assessment, is vital for comprehending and managing forest resources and ecosystems (Yun et al., 2024). The comprehensive use of multi-source data such as visible light, multispectral/hyperspectral, and LiDAR point clouds can provide a more comprehensive, multi-angle, and all-weather monitoring of plant growth conditions, however, it also brings more technical challenges. Deep learning is a powerful tool to meet these challenges employing more data and algorithms. Therefore, future research will focus on exploring superior feature extraction methods and ensemble strategies, and further introducing advanced technologies such as deep learning to achieve even higher precision in pests and diseases monitoring.

## Data Availability

The original contributions presented in the study are included in the article/supplementary material. Further inquiries can be directed to the corresponding author.
